# Needlestick-Associated Rocky Mountain Spotted Fever, Brazil

**DOI:** 10.3201/eid2604.191251

**Published:** 2020-04

**Authors:** Stefan Vilges de Oliveira, Álvaro A. Faccini-Martínez, Talita Emile Ribeiro Adelino, Ana Íris de Lima Duré, Amalia R.M. Barbieri, Marcelo B. Labruna

**Affiliations:** Universidade Federal de Uberlândia, Uberlândia, Brazil (S.V. de Oliveira);; Universidade Federal do Espírito Santo, Vitória, Brazil (Á.A. Faccini-Martínez);; Fundação Ezequiel Dias, Belo Horizonte, Brazil (T.E. Ribeiro Adelino, A.Í. de Lima Duré);; University of São Paulo, São Paulo, Brazil (A.R.M. Barbieri, M.B. Labruna)

**Keywords:** *Rickettsia*, rickettsial infections, *Rickettsia rickettsii*, needlestick injuries, tickborne diseases, zoonoses, bacteria, Brazil, Rocky Mountain spotted fever, RMSF, vector-borne infections

## Abstract

We report a fatal case of Rocky Mountain spotted fever (RMSF) in a man in Brazil without recent history of tick bites or environmental exposure. He received an accidental needlestick while working as a nurse. The nurse and his patient died. Both cases were confirmed as RMSF by molecular methods.

After viruses, bacteria are the most common infection risk in healthcare workers who have accidental exposure to blood or body fluids (*1*). Accidental exposures mainly occur from percutaneous injury or mucocutaneous contact (*1*).

*Rickettsia rickettsii* is the etiological agent of Rocky Mountain spotted fever (RMSF), a severe tickborne disease endemic to the Americas (*2*). In Brazil, RMSF is a notifiable disease, and 411 deaths were registered during 2007–2015 (*3*). Men from rural areas who were exposed to ticks in the environment around forests, rivers, and waterfalls accounted for >66% of cases (*3*). We report a fatal case of RMSF in a nurse who had no recent history of tick bite or environmental exposures.

In August 2018, two deaths in Minas Gerais state were classified as probable RMSF on the basis of clinical findings, including severe acute febrile syndrome. We retrospectively reviewed official report forms for the 2 cases (*4*). Case-patient A was a 74-year-old male farm worker from a rural area of Belo Horizonte municipality. On July 20, he began having symptoms of acute nonrash febrile syndrome, including myalgia, dysuria, and oliguria. He reported environmental exposure and an insect bite on his chest prior to onset of symptoms. He died on July 24 (Table). 

**Table:** Information about confirmed fatal case of needlestick-associated Rocky Mountain spotted fever and related source case in Minas Gerais state, Brazil, 2018*

Case-patient	Age, y/sex	Clinical signs and symptoms	Exposure factors	Date			qPCR (*gltA*)	Conventional heminested PCR (*ompA*)†
				Symptom onset	Serum collected	Death		
A, patient	74/M	Fever, myalgia, dysuria, oliguria	Environmental exposure to woods, rivers, waterfalls; report of insect bite	Jul 20	Jul 22	Jul 24	+	+
B, nurse	30/M	Fever, maculopapular rash, acute respiratory distress syndrome, shock, oliguria	No reported tick or insect bites or environmental exposures; accidental percutaneous needlestick injury associated with case-patient A on July 23	Jul 30	Aug 2	Aug 5	+	+

*Clinical and epidemiological data were retrieved from official spotted fever–rickettsiosis case forms collected for each patient by the Ministry of Health, Brazil (*4*). *gltA,* rickettsial citrate synthase gene; *ompA*, rickettsial outer membrane protein A gene; qPCR, quantitative PCR; +, positive. †All PCR amplicons were sequenced and confirmed a 100% identity with *Rickettsia rickettsii*.

Case-patient B was a 30-year-old man who had no history of recent travel, tick bites, or environmental exposures, nor did he own a dog. He was a nurse from the hospital where case-patient A was admitted. He reported an accidental percutaneous needlestick injury to his left thumb on July 23, after working with case-patient A in the hospital (Table). Following guidelines for biological hazards of healthcare workers in Brazil (*5*), clinicians collected blood from case-patient A and conducted serological tests for hepatitis B and C and HIV, all of which were negative. On July 30, case-patient B began having symptoms of acute febrile syndrome, including maculopapular rash, acute respiratory distress syndrome, shock, oliguria, thrombocytopenia, and leukopenia. Case-patient B died on August 5. Because RMSF was not suspected, neither case-patient received appropriate antimicrobial drugs. 

After reviewing the official spotted fever case reports, we suspected *R. rickettsii* infection in both cases. We tested serum samples collected on July 22 from case-patient A and on August 2 from case-patient B. We used a *Rickettsia* genus–specific quantitative PCR to amplify rickettsial *gltA* gene from the patients’ serum samples (*6*). Case-patient A had a cycle threshold value of 25.9 and case-patient B 35.3. We confirmed RMSF by using conventional heminested PCR protocol to amplify a 532-bp fragment of the rickettsial *ompA* gene, as previously described (*6*). Rickettsial DNA from the samples generated sequences with 100% identity to the corresponding *ompA* gene fragment of *R. rickettsii* (GenBank accession no. CP003305).

Besides the common transmission route through arthropod bite for infection, rare instances of *R. rickettsii* infection have been reported through accidental exposure in research laboratories or by percutaneous needlestick injuries in healthcare facilities. For instance, Johnson et al. described a series of 5 cases of laboratory-acquired RMSF cases in 1967, two of which occurred in workers who had accidental needlesticks involving a yolk-sac suspension of *R. rickettsii* (*7*). Both developed an acute febrile illness but were successfully treated with tetracycline (*7*). In another published case in a healthcare worker, a physician incurred a needlestick wound on his arm while assisting in the care of a patient with a presumptive diagnosis of RMSF. The patient died (*8*). The physician experienced sudden onset of a febrile illness 7 days after the puncture wound and a subsequent maculopapular rash. RMSF was confirmed by serological tests, and he was treated with oral tetracycline and recovered.

In accidental exposure, the risk of transmission varies according to the volume of blood inoculated and the number of infective agents in the inoculum (*1*). Median infective doses of rickettsiae are known to increase after endothelium destruction in severe cases of RMSF (*9*). In this case, when punctured with a needle, case-patient B probably was exposed to a high number of rickettsia released in the bloodstream of case-patient A just 1 day before his death. 

Our report highlights the importance of considering RMSF in patients with symptoms compatible with the disease and in healthcare workers caring for patients with undifferentiated fever in RMSF-endemic areas. Administering doxycycline before a rash occurs and within 5 days of symptom onset is crucial to patient recovery. Patients with a history of an arthropod bite, sudden onset of fever, and exposure in an endemic area should prompt clinicians to provide immediate treatment. Primary, secondary, and tertiary healthcare facilities educate and remind staff about RMSF and its associated signs and symptoms in patients.
